# Comparison of haemodynamic- and electroencephalographic-monitored effects evoked by four combinations of effect-site concentrations of propofol and remifentanil, yielding a predicted tolerance to laryngoscopy of 90%

**DOI:** 10.1007/s10877-020-00540-9

**Published:** 2020-06-03

**Authors:** J. P. van den Berg, A. R. Absalom, A. M. Venema, A. F. Kalmar, K. Van Amsterdam, L. N. Hannivoort, J. H. Proost, S. Meier, T. W. L. Scheeren, M. M. R. F. Struys, H. E. M. Vereecke

**Affiliations:** 1 Department of Anesthesiology, University Medical Center Groningen, University of Groningen, Hanzeplein 1, 9713 GZ Groningen, The Netherlands; 2 Department of Basic and Applied Medical Sciences, Ghent University, Ghent, Belgium; 3 Department of Anaesthesia and Reanimation, AZ Sint-Jan Brugge-Oostende AV, Brugge, Belgium

**Keywords:** Pharmacology, Interaction, Haemodynamic monitoring, Electroencephalographic monitoring

## Abstract

This prospective study evaluates haemodynamic and electroencephalographic effects observed when administering four combinations of effect-site concentrations of propofol (Ce_PROP_) and remifentanil (Ce_REMI_), all yielding a single predicted probability of tolerance of laryngoscopy of 90% (P_TOL_ = 90%) according to the Bouillon interaction model. We aimed to identify combinations of Ce_PROP_ and Ce_REMI_ along a single isobole of P_TOL_ that result in favourable hypnotic and haemodynamic conditions. This knowledge could be of advantage in the development of drug advisory monitoring technology. 80 patients (18–90 years of age, ASA I–III) were randomized into four groups and titrated towards Ce_PROP_ (Schnider model, ug⋅ml^−1^) and Ce_REMI_ (Minto model, ng⋅ml^−1^) of respectively 8.6 and 1, 5.9 and 2, 3.6 and 4 and 2.0 and 8. After eleven minutes of equilibration, baseline measurements of haemodynamic endpoints and bispectral index were compared with three minutes of responsiveness measurements after laryngoscopy. Before laryngoscopy, bispectral index differed significantly (p < 0.0001) between groups in concordance with Ce_PROP_. Heart rate decreased with increasing Ce_REMI_ (p = 0.001). The haemodynamic and arousal responses evoked by laryngoscopy were not significantly different between groups, but Ce_PROP_ = 3.6 μg⋅ml^−1^ and Ce_REMI_ = 4 ng⋅ml^−1^ evoked the lowest median value for ∆HR and ∆SAP after laryngoscopy. This study provides clinical insight on the haemodynamic and hypnotic consequences, when a model based predicted P_TOL_ is used as a target for combined effect-site controlled target- controlled infusion of propofol and remifentanil. Heart rate and bispectral index were significantly different between groups despite a theoretical equipotency for P_TOL_, suggesting that each component of the anaesthetic state (immobility, analgesia, and hypnotic drug effect) should be considered as independent neurophysiological and pharmacological phenomena. However, claims of (in)accuracy of the predicted P_TOL_ must be considered preliminary because larger numbers of observations are required for that goal.

## Introduction

**Fig. 1:**
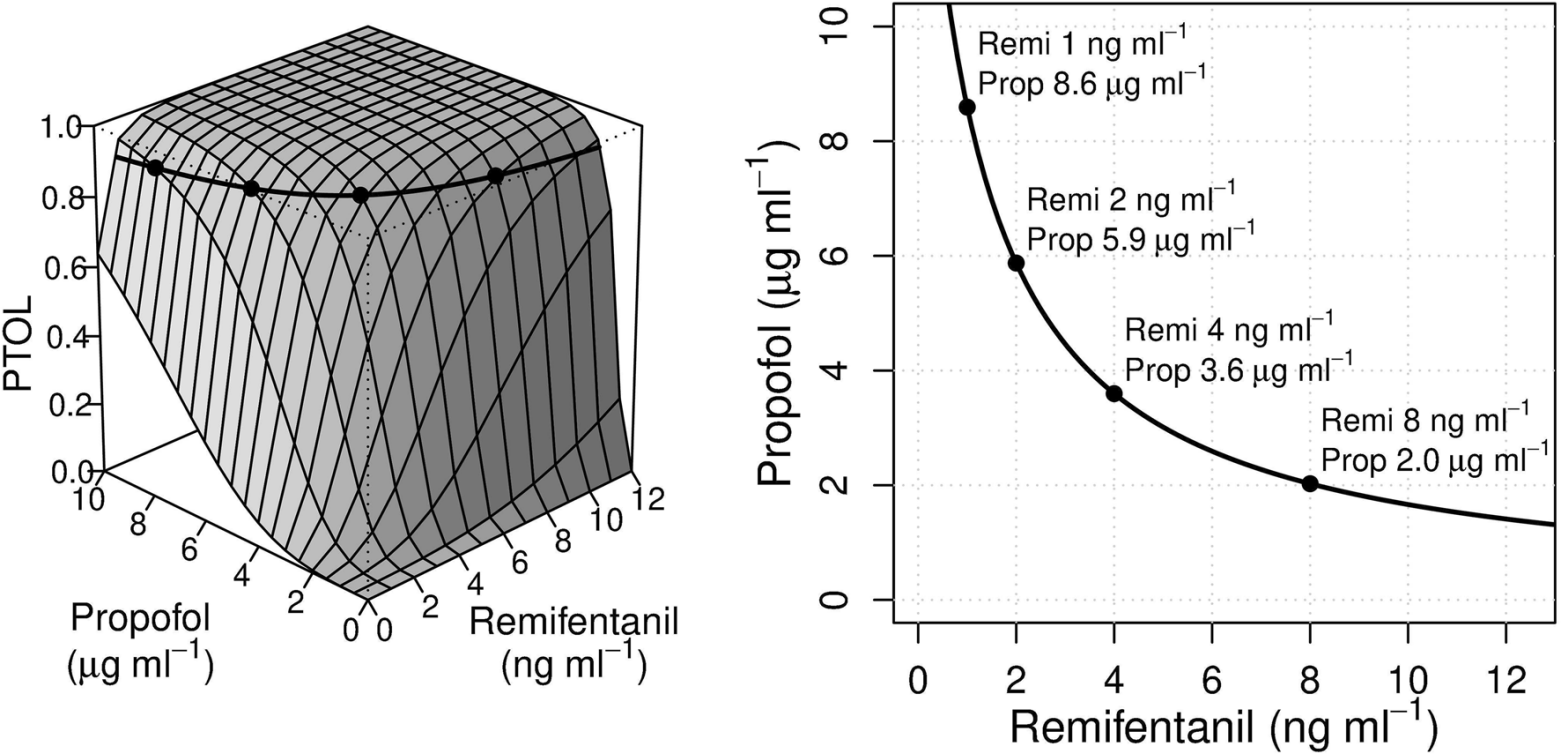
The left panel shows the three-dimensional response surface model of propofol and remifentanil based on the data of Bouillon et al. [3]. A response surface model is a mathematical description, visualized in a 3-dimensional graph depicting the effect (in this case, the probability of tolerance of laryngoscopy (P_TOL_)) (z-axis) evoked by combining two different drugs (respectively the effect-site concentration of propofol and remifentanil in the x and y axis). The thick line represents the P_TOL_ = 90% isobole, which is also depicted in the two-dimensional cross-sectional graph (right panel), representing the isoboles that connect all combinations of drugs that evoke identical effect. The bullets on the isobole represent the four drug combinations used in this study (A-D from left to right, respectively)

Pharmacodynamic drug interactions are classically described using isoboles, which are iso-effect lines that connect all combinations of drug concentrations resulting in an equivalent clinical drug effect [1]. Combining several isoboles in a three dimensional response surface model allows depiction of the total spectrum of the interaction between two drugs in relation to a specific effect of interest (Fig. 1). For example, the probability of tolerance to laryngoscopy (P_TOL_) can be predicted using response surface drug interaction models, for any combination of effect-site concentrations of propofol (Ce_PROP_) and remifentanil (Ce_REMI_) [2, 3], and it has been incorporated into commercially-available anaesthesia monitors, called advisory displays [1, 4]. The bedside availability of P_TOL_ creates opportunities for the clinician to improve the control over one of the main components of anaesthetic drug effect: the immobility in response to a noxious stimulus [1, 5, 6]. It now is clinically feasible to titrate different combinations of Ce_PROP_ and Ce_REMI_ while maintaining a constant effect estimated by P_TOL_. However, there is limited information whether titration towards various combinations of Ce_PROP_ and Ce_REMI_, that yield a similar P_TOL_, will be equally beneficial for the patient in view of electroencephalographic and haemodynamic effects, respectively representing the hypnotic and analgetic component of anaesthesia [2, 3, 6–11].

For electroencephalographically-derived indices monitoring hypnotic drug effect, such as the bispectral index (BIS)(Medtronic, Minneapolis, Minnesota, USA), Bouillon and coworkers predict that BIS will decrease in concordance with increases in Ce_PROP_, however this observation merits prospective confirmation [2]. In contrast, for commonly monitored haemodynamic endpoints, such as heart rate (HR), systolic arterial blood pressure (SAP), stroke volume (SV), cardiac index (CI), systemic vascular resistance (SVR), and the systemic vascular resistance index (SVRI), it is striking that the interaction between Ce_PROP_ and Ce_REMI_ has not been described equally thorough using isoboles or response surface interaction models.

Therefore, this study explores the clinical consequences of a P_TOL_ guided titration using effect-site controlled infusion of propofol and remifentanil, in order to provide helpful insight on the haemodynamic and hypnotic consequences of this titration concept, using clinical advisory display monitors. The primary purpose was to compare the haemodynamic and electroencephalographic effects observed between four different combinations of Ce_PROP_ and Ce_REMI_, all yielding a P_TOL_ of 90% as estimated by Bouillon et al. [3], both during steady-state conditions (before laryngoscopy), as well as in response to a laryngoscopy. For all measured endpoints, we hypothesize that no significant difference would be found between groups because the clinical condition is estimated to be equipotent for P_TOL_.

As commercially advisory monitors become available as a monitoring tool to guide titration of combinations of drugs towards equipotency for PTOL [1], this study is a first attempt to explore the subsequent haemodynamic consequences of such actions in a prospective study. It must be demonstrated in a standardised and reproducible way that it is clinically safe to adjust drug administration according to the advice provided by these new advisory monitors.

## Materials and methods

Following Ethics Committee approval (University Medical Center Groningen, University of Groningen, Groningen, The Netherlands, METc 2013/267) and clinical trial registration (ClinicalTrials.gov, #NCT02067936, principal investigator: H.E.M. Vereecke, Date of Registration: February 20th 2014, https://goo.gl/z23qJG) prior to patient enrolment, written informed consent was obtained from 80 patients (18–90 years of age, ASA I–III) scheduled for general anaesthesia for elective neurosurgical or maxillofacial surgical procedures. Exclusion criteria were BMI exceeding 35 kg∙m^−2^, allergies for either propofol and/or remifentanil, overt signs of alcohol or drug abuse, diseases of the central nervous system, hepatic disease (i.e. Child B or higher), the use of drugs acting on the central nervous system, and the use of α-or β-receptor agonists or antagonists. Patients suspected for having a difficult airway were excluded to ensure a safe mask ventilation throughout the measurements.

### Study design

The study was designed as a prospective double-blind, randomized, interventional trial. All measurements and study interventions were performed in a quiet operating room, before surgery commenced. After screening for eligibility and informed consent, 80 patients were randomized into four groups of 20 participants each, using sealed envelopes drawn at random. Patients received 1000 mg paracetamol as premedication. No benzodiazepine or other sedative drug was administered to the patient prior to the procedure.

The anaesthesiologist who performed mask ventilation, laryngoscopy, and the observation of motor response was blinded to group allocation. If any airway problem occurred at any time during the study, the anaesthesiologist could decide to un-blind the randomization and take all necessary measures. Such patients were excluded from further data analysis and replaced. Administration of the drugs and data collection was performed by an (unblinded) researcher who operated a laptop running RUGLOOP II software (Demed Engineering, Temse, Belgium), a data collection program which also controls the target-controlled infusion pumps.

An intravenous line was inserted in a large forearm vein. Electrocardiography (ECG), pulse-oximetry (SpO_2_), and intermittent non-invasive blood pressure (NIBP) was measured using the Philips Intellivue MP70 patient monitor (Philips Healthcare, Eindhoven, The Netherlands). CI, SV, SVI and SVRI were measured continuously by the ccNexfin Monitor (BMEYE, Amsterdam, Netherlands; now Edwards Lifesciences, Irvine, CA, USA). Measurements were performed using a non-invasive ccNexfin finger cuff attached to the third digit of the contralateral hand of the NIBP measurements. The heart reference system (HRS™) of Nexfin® was positioned at mid-axillary level in the supine positioned patient to calibrate for hydrostatic pressure differences between the position of the finger and the level of the heart.

Bispectral index was measured using a unilateral BIS Quatro Sensor (and a BIS Vista monitor (all: Medtronic, Dublin, Ireland), smoothing delay time was set at 15 s with data storage every second. BIS and suppression ratio were compared between groups.

### Study drug administration

Propofol 2% (Braun, Melsungen, Germany) and remifentanil 50 µg⋅ml^−1^ (GlaxoSmithKline BV, Zeist, The Netherlands) were both administered by means of RUGLOOP II, a data collection and drug delivery software that runs on a PC (Penta Hercules Medical ETX, Penta GmbH, Mönchengladbach, Germany) connected to infusion pumps (Asena GH, Becton Dickinson, San Diego, California, USA) and monitoring devices through USB ports, RS-232 interface and LAN connectivity. The software collects all data in a time-synchronized way. The pumps administer propofol and remifentanil using an effect-site controlled target-controlled infusion with maximum infusion rates of 600 and 1200 ml⋅h^−1^ respectively. For calculating Ce_PROP_ and Ce_REMI_ the pharmacokinetic-dynamic models of Schnider and Minto respectively were applied [7, 8]. For the Schnider model, the elimination equilibration constant of the effect-site (ke0) was fixed to 0.456 min^−1^. These models are identical to those used by Bouillon and coworkers for the interaction model development [3].

### Study procedure

After checking signal quality of all measurements, and before any drug was administered, patients were asked to close their eyes for 2 min for registration of awake measurements while breathing 100% oxygen through a gently applied face mask. Next, propofol and remifentanil infusions were started simultaneously, targeted to one out of four equipotent drug combinations of Ce_REMI_ and Ce_PROP_ (see Fig. 1). These combinations were determined as follows: First, four values of Ce_REMI_ were chosen over a wide clinically relevant range, i.e. 1, 2, 4 and 8 ng⋅ml^−1^ for groups A, B, C and D, respectively. The corresponding Ce_PROP_ resulting in P_TOL_ = 90% was obtained from the sigmoidal response function for a dichotomous effect [2, 9, 10].

1 $${\text{P}}_{{{\text{TOL}}}} = { }\frac{{{\text{U}}^{{\upgamma }} }}{{1 + {\text{U}}^{{\upgamma }} }}$$

where γ is the slope parameter, representing the steepness of the concentration-effect relationship, and U is the combined potency of the drugs according to

2 $${\text{U}} = { }\frac{{{\text{Ce}}_{{{\text{PROP}}}} }}{{{\text{Ce}}50_{{{\text{PROP}}}} }}{*}\left[ {1 + \frac{{{\text{Ce}}_{{{\text{REMI}}}} }}{{{\text{Ce}}50_{{{\text{REMI}}}} }}} \right]$$

where Ce50_PROP_ is the effect-site concentration of propofol resulting in P_TOL_ = 0.5 if given alone, and Ce50_REMI_ is the Ce_REMI_ that results in an increase of U by a factor 2 or an apparent decrease of Ce50_PROP_ by 50%. The model parameters of the Bouillon study were taken from Table 1 of the article by Hannivoort et al. [10]: Ce50_PROP_ = 8.48 µg⋅ml^−1^, Ce50_REMI_ = 1.16 ng⋅ml^−1^ and γ = 3.46. It follows from Eq. 1 that U = 1.887 for P_TOL_ = 90%. For each of the four remifentanil concentrations, the corresponding propofol concentration was calculated from Eq. 2, resulting in the previously mentioned drug dose combinations.

**Table 1:** Patient characteristics and comparison between haemodynamic- and electroencephalographic baseline measurements. Baseline heart rate was different between groups (p < 0.000), as was BIS between groups (p < 0.001)

	Group A Remifentanil 1 ng⋅ml^−1^; propofol 8.6 μg ⋅ml^−1^	Group B Remifentanil 2 ng⋅ml^−1^; propofol 5.9 μg ⋅ml^−1^	Group C Remifentanil 4 ng⋅ml^−1^; propofol 3.6 μg ⋅ml^−1^	Group D Remifentanil 8 ng⋅ml^−1^; propofol 2.0 μg ⋅ml^−1^	
A: Patient characteristics					
Age (years); Mean (SD) Range []	50 (± 13.7) [46]	52 (± 13.1) [50]	51 (± 17.5) [56]	58 (± 10.7) [33]	
Gender (m/f)	10/10	10/10	10/10	12/8	
BMI (kg⋅m^−2^); Median [range]	26 [23–29]	26 [24–28]	26 [24–26]	26.5 [23–28]	
B: Median baseline values Range [P25-P75]					
					p
Heart rate (beats ⋅ min^−1^)	71^#^ [60–73]	68^$ %^ [63–72]	60^$^ [56–63]	60^# %^ [56–62]	0.003
Systolic blood pressure (mmHg)	94 [84–101]	90 [79–105]	97 [87–101]	104 [80–108]	0.629
Stroke volume index (ml ⋅ m^−2^)	44 [36–45]	39 [35–46]	42 [34–46]	40 [34–48]	0.947
Systemic vascular resistance index (dyn ⋅ s ⋅ cm^−5^)	2104 [1804–2337]	2151 [1902–2544]	2226 [2063–2556]	2665 [2021–3095]	0.333
Cardiac Index (L ⋅ min^−1^ ⋅ m^−2^)	2.60 [2.38–3.23]	2.55 [2.30–3.20]	2.35 [2.19–2.62]	2.25 [1.98–2.80]	0.162
BIS	25 ^@ ^^ [23–27]	30 ^+ §^ [25–40]	44 ^@ + £^ [41–55]	64 ^^ § £^ [59–72]	< 0.001

^#^p = 0.004; $p = 0,045; %, @, ^,+, § and £ p < 0.0001

Once Ce_PROP_ and Ce_REMI_ reached the desired target, eleven minutes of equilibration time ensures minimal prediction errors of the applied models during the observations [11]. Subsequently, one minute of baseline measurement was performed followed by a laryngoscopy, with an aim to visualize the larynx and thereby exert a standardized noxious stimulus, for a duration of 10 s. The time point of ‘laryngoscopy’ originally was registered as the moment of full traction on the laryngoscope. We noticed during the conduct of the study that the manipulations to start laryngoscopy (such as removal of the face mask and oral introduction of the laryngoscope) may already have triggered haemodynamic, motor, or electroencephalographic responses in some cases, several seconds before maximum traction was reached. For the analysis of data, we pragmatically defined the “start of laryngoscopy” as a time point 30 s before full traction was applied. After laryngoscopy, another three minutes of data registration were maintained to observe the presence or absence of movement and haemodynamic or electroencephalographic measurements. Motor response was defined as movement of extremities, opening of eyes, gag reflex or coughing within three minutes after the initial manipulations for starting laryngoscopy. After the three minutes observation of response, the study ended, and regular care was resumed.

### Safety precautions

In case of unsustainable respiratory inhibition or apnea during induction or during the equilibration time, gentle airway manoeuvres (e.g. adjustment of the face mask, chin lift, hyperextension or pillow adjustments) were performed to facilitate mask-bag ventilation. In case of difficulties to obtain an open airway, or haemoglobin oxygen desaturation, an oropharyngeal airway was introduced. If respiratory escape manoeuvres caused a motor, haemodynamic, or arousal response during the measurements of the pre-stimulation baseline minute (minute 10 to 11) the patient was excluded from analysis, due to the lack of steady state conditions. In case of bradycardia below 40·min^−1^ or a decrease in mean arterial pressure below 50 mmHg, respectively 0.5 mg of atropine or 5 mg of ephedrine, was administered as rescue medication. The number of rescue interventions and the total dose of atropine and ephedrine were also registered for comparison between groups.

### Statistics and data analysis

For the primary haemodynamic and electroencephalographic endpoints, no previous data was available to make reasonable assumptions for an a-priori power calculation of the sample size required. We thus pragmatically chose to include 20 patients per group, based on our previous experiences in pharmacodynamic studies. In this clinical evaluation study, we accepted the validity and accuracy of the published values for P_TOL_[3].

All collected data from different storage systems was synchronized using an algorithm in R (R, The R foundation for statistical computing, Vienna, Austria), after rejecting obvious false values (e.g. negative blood pressure). The algorithm defined a short-lasting (< 1 sec) difference of 30% between two subsequent time points (1 s interval) as an artefact. BIS-measurements with a signal quality index < 50 were removed. All raw data (after artefact rejection) of the haemodynamic and BIS-measurements were plotted from the baseline minute to three minutes after laryngoscopy. We also plotted SR as measured by BIS.

Significant difference was defined when p < 0.05. Baseline characteristics were compared using ANOVA tests or a non-parametric Kruskal Wallis test with Nemenyi post hoc test, depending on normality of the distribution, which was tested using the Shapiro–Wilk test. After testing for normality, haemodynamic variables, (HR, SAP, CI, SVI, SVRI) and BIS were compared between groups, both at baseline and in response to laryngoscopy. The median and range of the baseline data were compared using a Kruskall-Wallis test.

To allow a comparison of the magnitude of haemodynamic (∆HR, ∆SAP, ∆CI, ∆SVI, ∆SVRI) and arousal responses (∆BIS) evoked by laryngoscopy between groups, the difference between the median of the baseline values and the maximum value observed during the response period was calculated for all endpoints and for each subject, and then averaged (median ± standard deviation) for the population. A Kruskall-Wallis test was used to test for significant differences between groups.

Whenever the individual ∆HR, ∆SAP, ∆CI, ∆SVI, ∆SVRI exceeded 20% of the baseline value, we defined that case as a “haemodynamic responder” and compared the number of responders between groups. A threshold of 20% was chosen as it is a commonly used definition of perioperative hypotension in current literature [12–14]. For ∆BIS, we also counted the number of arousal responses in a similar fashion, but used a 30% threshold instead. Changes in BIS < 20% rarely trigger a therapeutic intervention because fluctuations of that magnitude over time are common for BIS [15].

## Results

**Fig. 2:**
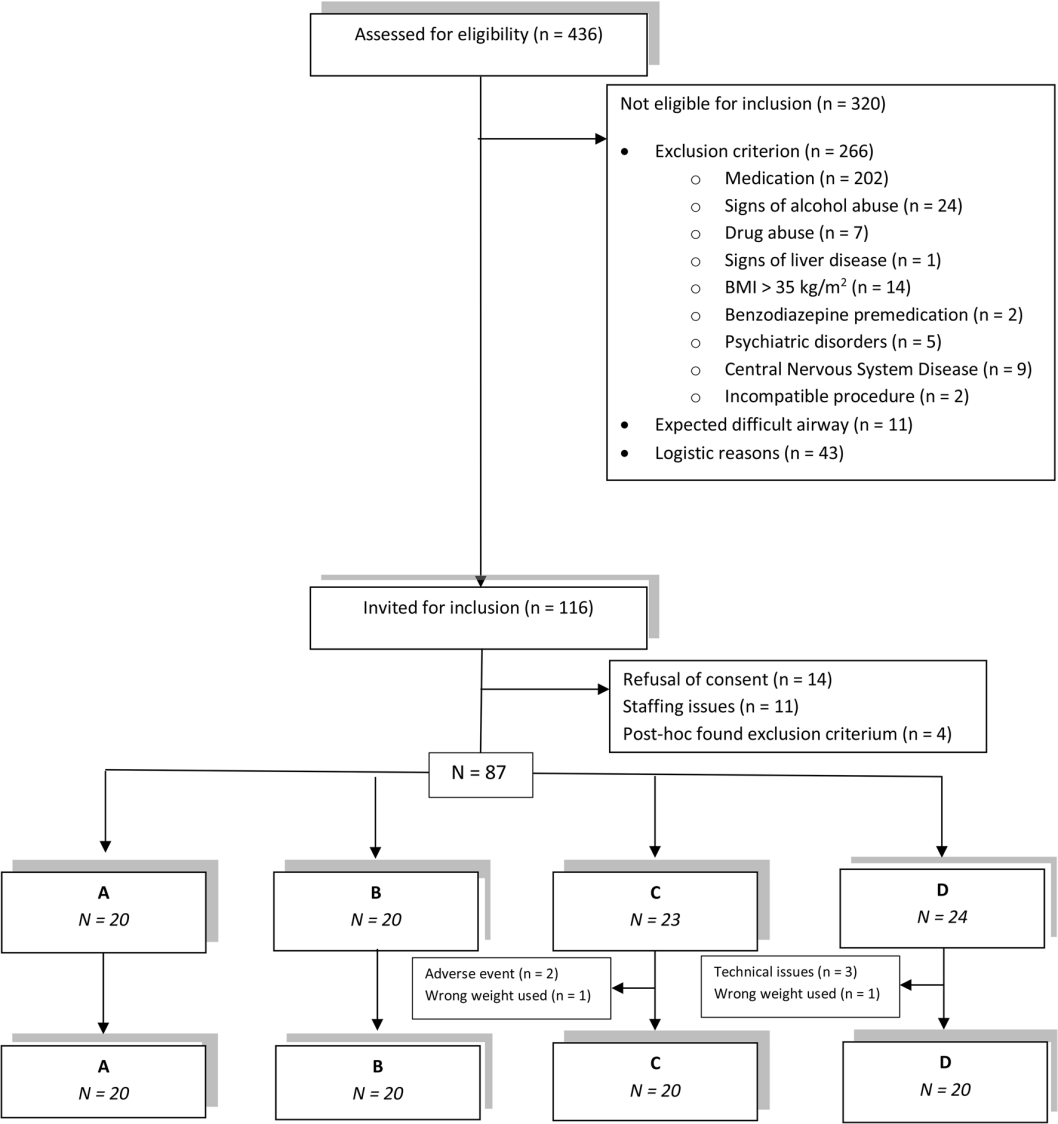
CONSORT diagram

An overview of the screenings/inclusion/exclusions can be found in the CONSORT diagram (Fig. 2). Patient characteristics are described in Table 1. No serious adverse events occurred during the conduct of the study. 87 patients were randomized to the four groups. In groups C and D, 3 and 4 patients were replaced, respectively. The remaining 80 patients were included in the data analysis. The patient characteristics were comparable between groups (Table 1, panel A).

**Fig. 3:**
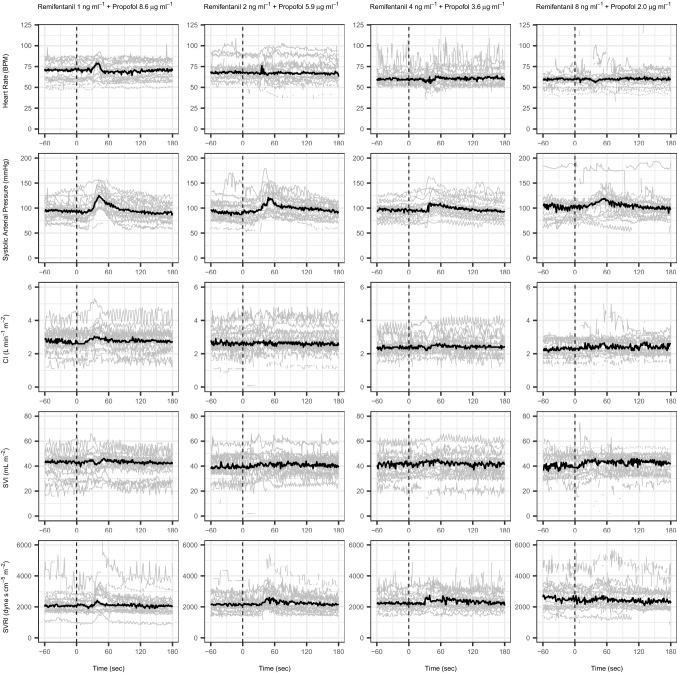
The evolution of the observed haemodynamic variables between − 60 to + 120 s from the start of laryngoscopy (Time = 0). The grey lines represent the results for each individual patient. The black line is the population median value. Group A (remifentanil 1 ng ⋅ ml^−1^ and propofol 8.6 µg ⋅ ml^−1^), Group B (remifentanil 2 ng ⋅ ml^−1^ and propofol 5.9 µg ⋅ ml^−1^), Group C (remifentanil 4 ng ⋅ ml^−1^ and propofol 3.6 µg ⋅ ml^−1^), and Group D (remifentanil 8 ng ⋅ ml^−1^ and propofol 2.0 µg ⋅ ml^−1^)

**Fig. 4:**
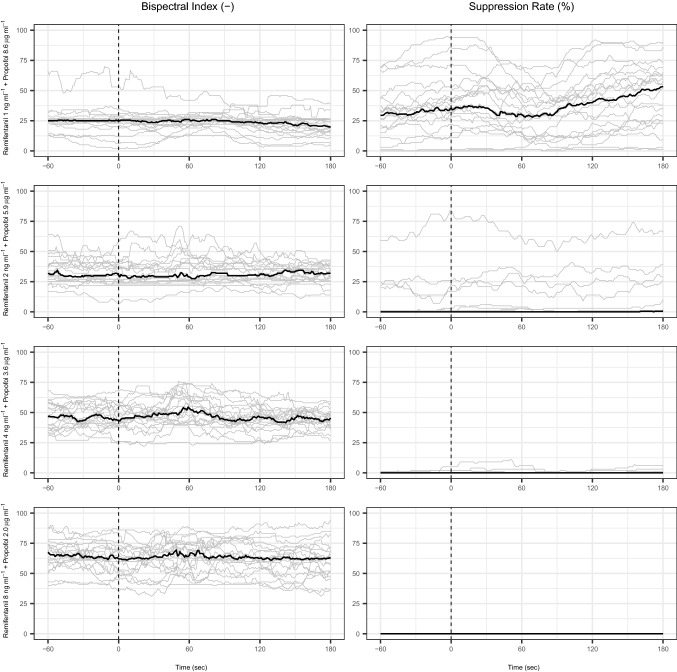
The evolution of the observed electroencephalographic variables between − 60 to + 120 s from the start of laryngoscopy (Time = 0). The grey lines represent the results for each individual patient. The black line is the population median value. Group A (remifentanil 1 ng ⋅ ml^−1^ and propofol 8.6 µg ⋅ ml^−1^), Group B (remifentanil 2 ng ⋅ ml^−1^ and propofol 5.9 µg ⋅ ml^−1^), Group C (remifentanil 4 ng ⋅ ml^−1^ and propofol 3.6 µg ⋅ ml^−1^), and Group D (remifentanil 8 ng ⋅ ml^−1^ and propofol 2.0 µg ⋅ ml^−1^). BIS SR = suppression ratio as measured by the bispectral index monitor

In Figs. 3 and 4, the haemodynamic and electroencephalographic endpoints, respectively, are plotted over time from the baseline measurements till the end of the response observation time. These figures give an impression of the population variability at baseline for each studied measurement, and the magnitude of the respective responses to laryngoscopy.

We observed 2, 1, 4, and 8 motor responders after laryngoscopy in group A, B, C, and D, respectively. The higher number of responders in group D may be related to several patients who did not reach a sufficient level of hypnotic drug effect before laryngoscopy, as also reflected in the high BIS values. Even the removal of the face mask resulted in return of responsiveness for some patients of group D.

Table 1 panel B shows the statistical results for the comparison of the baseline measurements between groups. HR at baseline was significantly higher in groups A and B versus C and D (p < 0.000): median HR (25–75% range) for groups A, B, C and D were 71 (59–73), 68 (63–72), 60 (56–63) and 60 (56–62) beats⋅min^−1^, respectively. Median CI was 0.3 L⋅min^−1^ higher in groups A and B compared to C and D (not statistically significant, p = 0.162). Median SAP, CI, SVI and SVRI were not significantly different at baseline between groups.

Table 2 shows the results of the magnitude of the haemodynamic and arousal responses after laryngoscopy (∆HR, ∆SAP, ∆CI, ∆SVI, ∆SVRI, ∆BIS). The median haemodynamic responses evoked by laryngoscopy were moderate and not significantly different between groups for all measured variables. Group C had the lowest median value for ∆HR and ∆SAP.

**Table 2:** Comparison of difference between the median value before versus the maximum value after laryngoscopy for each variable. BIS was different between groups (p = 0.006), as was systolic blood pressure (p = 0.03)

Δpre-post stimulation (MD [P25-P75]	Group A Remifentanil 1 ng⋅ml^−1^; propofol 8.6 μg ⋅ml^−1^	Group B Remifentanil 2 ng⋅ml^−1^; propofol 5.9 μg ⋅ml^−1^	Group C Remifentanil 4 ng⋅ml^−1^; propofol 3.6 μg ⋅ml^−1^	Group D Remifentanil 8 ng⋅ml^−1^; propofol 2.0 μg ⋅ml^−1^	P
Δ Heart rate (beats ⋅ min^−1^)	10 [7–14]	10 [4–15]	6 [3–16]	8 [4–30]	0.498
Δ Systolic blood pressure (mmHg)	32 [21–40]	30 [18–45]	19 [8–32]	21 [11–40]	0.153
Δ Stroke volume index (ml ⋅ m^−2^)	7 [5–9]	8 [5–9]	6 [4–8]	5 [4–8]	0.750
Δ Systemic vascular resistance index (dyn ⋅ s ⋅ cm^−5^)	485 [269–1143]	849 [455–1313]	532 [412–1029]	800 [329–3726]	0.558
Δ Cardiac index (L ⋅ min^−1^ ⋅ m^−2^)	0.55 [0.38–0.72]	0.40 [0.38–0.70]	0.50 [0.2–0.72]	0.40 [0.3–1.1]	0.799
Δ BIS	3# [2–7]	7 [3–11]	11# [7–17]	8 [4–12]	0.011

^#^p = 0.005

We identified 5 (25%), 6 (30%), 7 (35%), and 9 (45%) HR responders in groups A, B, C, and D, respectively, who had a 20% increase of HR evoked by laryngoscopy.

For ∆SAP, groups A and B contained almost double the number of responders of groups C and D (16, 17, 8, and 9 for groups A, B, C, and D, respectively).

After induction and during the equilibration phase, respectively 7, 4, 10, and 5 out of 20 patients in group A, B, C, and D needed rescue medication for hypotension being one or two administrations of ephedrine 5 mg, before baseline measurements were performed. No administration of ephedrine was needed during the steady state measurements. No atropine was administered as a rescue for bradycardia.

For BIS at baseline, groups A and B showed significantly lower BIS (p < 0.000) at baseline compared to groups C and D (Table 1, Panel B), which is consistent with the higher Ce_PROP_ in groups A and B. Also, groups A and B contained the highest number of cases with SR levels > 5% (Fig. 4). In group C, we observed the highest number of patients with continuous BIS values that remained within the desired range (between 40 and 60). (Fig. 4). Only 3 out of 20 patients showed minimal burst suppression patterns (SR < 5%) at baseline in group C.

For the BIS response to laryngoscopy, only group A showed a statistically significant lower ΔBIS compared to group C (p = 0.011), which may be related to the high incidence of SR > 5% at baseline, indicating excessive hypnotic drug effect in group A. For all other groups ΔBIS was indistinguishable between groups. The number of BIS arousal responses (> 30% increase after laryngoscopy) was respectively 25, 40, 40, and 10% for groups A to D. The low incidence of arousal in group A and D are probably both biased due to respectively the high incidence of SR > 5% in group A and the high baseline BIS values in group D that cannot rise another 30%.

## Discussion

This study explores the haemodynamic and hypnotic effects evoked by four different combinations of Ce_PROP_ and Ce_REMI_, all yielding an identical predicted P_TOL_ according to the Bouillon interaction model. P_TOL_ is a theoretical -population derived- metric for immobility in response to a noxious stimulus and has been integrated in clinically-available advisory monitors for drug titration during anaesthesia, however, the clinical usefulness still needs further prospective validation. In this clinical evaluation study, we accepted the validity and accuracy of the published values for P_TOL_[3]. As such, any claim of (in)accuracy of the predicted P_TOL_ must be considered preliminary because larger numbers of observations are required for that goal. However, as commercialized advisory screens are already clinically available to allow titration towards this population derived prediction of P_TOL_, our current dataset does provide valuable information on the haemodynamic and hypnotic consequences of such deliberately combined drug titration using effect-site controlled target-controlled infusion [5, 16].

We did find that titration towards the four theoretically equipotent combinations of Ce_PROP_ and Ce_REMI_ (for predicted P_TOL_ = 90%) does result in moderate -but clinically relevant- differences of haemodynamic and hypnotic drug effects between groups.

Before laryngoscopy, similar SAP, CI, SVI, and SVRI was found in all groups except for heart rate, which was moderately (but statistically significant) lower in groups with higher Ce_REMI_ concentrations. This is in concordance with a study of Hayashi and coworkers showing a cut-off point for heart rate reduction at remifentanil concentrations around 3.5 ng⋅ml^−1^[17].

The apparent similar haemodynamic state between groups before laryngoscopy could only be achieved after a variable number of patients reached a safety threshold during the non-steady state induction of anaesthesia. They needed rescue treatment with ephedrine at variable timepoints. We were not able to identify any causal relationship between the occurrence of such events and group randomization, neither for the number of rescue treatments nor for the total dose of ephedrine administered. This observation may challenge the commonly accepted notion that high levels of opioids during balanced anaesthesia result in more stable haemodynamic conditions. It merits further research towards the immediate haemodynamic effects evoked by the non-steady state induction of anaesthesia, using effect-site controlled target-controlled infusion. For this study pharmacological steady-state conditions were required, which was only reached after an equilibration period of 11 min. As such, we cannot report on observations during non-steady state conditions.

After laryngoscopy, one would expect the lowest haemodynamic response in groups with higher Ce_REMI_, however, the median haemodynamic responses (∆HR, ∆SAP, ∆CI, ∆SVI, ∆SVRI) were moderate in severity and not significantly different between groups (Table 2). The number of patients that showed a > 20% difference in baseline HR was similar for groups A, B, and C. Group D had the highest incidence (45%) of HR responses. This finding may be a biased by the substantial number of patients with high BIS (> 60) in group D, suggesting an insufficient hypnotic drug effect. Indeed, several patients immediately opened their eyes once the face mask was removed and before laryngoscopy could be initiated. For ∆SAP, groups A and B contained almost double the number of responders of groups C and D, which is concordant with the higher Ce_REMI_ in groups C and D.

BIS showed significant differences in baseline measurements with several cases of high SR > 5%, indicating an excessive hypnotic drug effect in groups A and B (Fig. 4). In contrast, group D included many cases of BIS above the upper limit threshold of 60 (increasing the risk for conscious perception). Therefore, BIS appears to be inversely related to Ce_PROP_, which agrees with earlier studies [10, 18]. An arousal response of BIS evoked by laryngoscopy may be a sign of inadequate analgesic effect and is associated with increased risks for unintentional conscious perception [19]. The magnitude of arousal, measured by BIS, was small in our study and not significantly different between groups (Table 2).

Our data allows to identify whether some combinations of Ce_PROP_ and Ce_REMI_ evoke more favourable haemodynamic conditions, when evaluating all effects together. We found that in group C, during baseline measurements, only a moderate decrease in HR and no significant difference in SAP was observed compared to other groups. Group C also contained the highest number of patients with an adequate BIS range before laryngoscopy while the absence of high SR suggests absence of overdosing of hypnotic drug effect. Group C also had the lowest median value for ∆HR and ∆SAP evoked by laryngoscopy. We observed 4 motoric responders out of 20 in group C, which is an acceptable clinical result, considering the predicted P_TOL_ = 90% and its corresponding confidence interval (not published post-hoc analysis). However, the latter conclusion must be interpreted cautiously due to the insufficient power of our study to validate the accuracy of P_TOL_ as a metric of drug effect.

In conclusion, this study provides clinical insight on the haemodynamic and hypnotic consequences, when a model based predicted P_TOL_ is used as a target for combined intravenous administration of propofol and remifentanil, using effect-site controlled target-controlled infusion pumps. Heart rate and bispectral index were significantly different between groups despite a theoretical equipotency for P_TOL_, suggesting that each component of the anaesthetic state (immobility, analgesia, and hypnotic drug effect) should be considered as independent neurophysiological and pharmacological phenomena.
